# Does Statin Therapy Reduce the Risks of Mortality and Major Adverse Cardiac and Cerebrovascular Events in Young Adults with End-Stage Renal Disease? Population-Based Cohort Study

**DOI:** 10.3390/jcm10102097

**Published:** 2021-05-13

**Authors:** Ya-Lien Cheng, Huang-Yu Yang, Chao-Yi Wu, Chung-Ying Tsai, Chao-Yu Chen, Ching-Chung Hsiao, Hsiang-Hao Hsu, Ya-Chung Tian, Chieh-Li Yen

**Affiliations:** 1 Kidney Research Center, Department of Nephrology, Chang Gung Memorial Hospital, Linkou Branch, Taoyuan 33305, Taiwan; yolien0205@gmail.com (Y.-L.C.); hyyang01@gmail.com (H.-Y.Y.); cytsai0616@cgmh.org.tw (C.-Y.T.); Chaoyuclaire@gmail.com (C.-Y.C.); colinhua0123@gmail.com (C.-C.H.); hsianghao@gmail.com (H.-H.H.); dryctian@cgmh.org.tw (Y.-C.T.); 2 College of Medicine, Chang Gung University, Taoyuan 33305, Taiwan; 3 Division of Rheumatology, Allergy and Immunology, Chang Gung Memorial Hospital, Taoyuan 33305, Taiwan; joywucgu@hotmail.com

**Keywords:** statin, dialysis, ESRD, young adult, cardiovascular

## Abstract

Among hemodialysis patients aged more than 40 years old, previous large-scale studies showed statin treatment had no effect on reducing cardiovascular adverse events. However, young-adult-onset end-stage renal disease (ESRD) patients have different physicosocial factors compared to older ESRD patients. The benefit of statins in such a specific group has not been well evaluated. Through the use of Taiwan’s National Health Insurance Research Database (NHIRD), young adult patients aged 20–40 with incident ESRD requiring permanent dialysis between 1 January 2003 and 31 December 2015 were identified. The enrollees were further divided into two groups depending on whether they received statin therapy for more than 90 days (statin group) or never received any statin (nonstatin group) in the first year after initiation of dialysis. Propensity score weighting (PSW) was used to balance the baseline characteristics between the two groups. After PSW, the statin group (*n* = 771) exhibited a higher rate of major adverse cardiac and cerebrovascular events (MACCEs) (2.65% vs. 1.44%, hazard ratio (HR): 1.87, 95% confidence interval (CI): 1.43–2.45), and acute myocardial infarction (1.51% vs. 0.30%, HR: 5.34, 95% CI: 3.40–8.39) compared to the nonstatin group (*n* = 1709). The risk of all-cause mortality, cardiovascular (CV) death. and stroke did not significantly differ between the two groups. Similar to older patients, this study demonstrated that statin therapy cannot offer any protective effects in reducing CV outcomes among young adult ESRD patients undergoing dialysis.

## 1. Introduction

Considerable research has confirmed that statins can considerably reduce the incidence of major adverse cardiovascular events and risk of mortality in high-risk patients, such as those with hyperlipidemia and diabetes [1,2,3,4,5]. Statins offer significant cardiovascular (CV) benefits by effectively reducing circulating low-density lipoprotein (LDL) cholesterol through the inhibition of hepatic hydroxymethylglutaryl (HMG) coenzyme A (CoA) reductase, leading to the stabilization or even regression of atherosclerotic plaque. However, several large-scale randomized controlled trials on statin treatment [6,7,8] and high-quality meta-analyses [9] have reported that statin treatment did not provide significant CV protective effects in patients with end-stage renal disease (ESRD) as they did in traditional high-risk groups despite their marked LDL-cholesterol-lowering ability. This lack of CV benefit has been attributed to long-term comorbidities, such as diabetic vasculopathy and hyperlipidemia in early chronic kidney disease (CKD). These chronic vascular conditions are often irreversible and cannot be improved by statin treatment during ESRD [10,11]. CV mortality among the ESRD population has also been primarily attributed to nonatherosclerosis CV events, such as arterial calcification due to hyperphosphatemia and hypercalcemia and left ventricular hypertrophy (LVH) due to poor fluid control after dialysis initiation. These nonatherosclerosis CV events respond poorly to statin-induced LDL reduction [11,12,13,14]. However, why statin treatment fails to provide benefits in patients with ESRD remains unclear.

Most previous studies enrolled only older patients with ESRD. The 4D study recruited patients with ESRD aged 18 to 80 years, with an average age of 65.7 years [6]. The AURORA and SHARP studies recruited patients receiving dialysis aged 50 to 80 and >40 years, respectively [7,8]. Young adults with ESRD have been reported to demonstrate distinct characteristics in the incidence and prevalence of chronic illnesses. In a retrospective study, compared with older patients, young adults with ESRD often had a shorter duration of pre-existing, non-dialysis-requiring CKD prior to ESRD, resulting in a lower CV disease burden over time [15]. Moreover, the ESRD etiology, comorbidities, and performance status of younger patients receiving dialysis are different from those of older patients [16,17]. According to epidemiological studies, glomerulonephropathy, hypertension, genetic and metabolic diseases, and congenital anomalies of the kidney and urinary tract—rather than diabetes, tumor, nephrolithiasis, or other chronic systemic diseases—are the leading causes of ESRD in young adults [18,19]. However, the CV mortality rates of younger and older patients receiving dialysis are similar. Nearly 40% of deaths were attributable to CV causes among young adults aged 22 to 29 years with incident ESRD [15,20]. The risk of CV mortality in young adults with incident ESRD is 143 to 500 times higher than that of age-matched individuals in the general population [21]. Thus, potential treatment strategies to reduce the likelihood of CV events among this population are worth investigating.

Few studies on the clinical outcomes and efficacy of statin treatment in young adults with incident ESRD undergoing dialysis have been conducted. We assume that statin treatment may provide significant benefits in young adults with ESRD because they have not yet been affected by aging, have fewer systemic chronic illnesses, and have a shorter duration of the CKD burden. In this study, we employed Taiwan’s National Health Insurance (NHI) Research Database (NHIRD), which contains nationwide population-based information, to determine whether statins can reduce the mortality and major adverse cardiac and cerebrovascular event (MACCE) risks in patients aged between 20 and 40 years with ESRD and requiring permanent dialysis.

## 2. Materials and Methods

### 2.1. Data Source

Patient data were obtained from Taiwan’s NHIRD. In 1995, Taiwan launched the NHI program, a nationwide, single-payer, compulsory health care system covering approximately 99.8% of Taiwan’s population (nearly 23.37 million) since 1997. The NHIRD contains comprehensive information about insured patients’ health care, including disease diagnoses, outpatient visits, hospitalizations, procedures, medication prescriptions, and specific conditions. The diagnosis of diseases in the NHIRD is based on the International Classification of Diseases, 9th Revision, Clinical Modification (ICD-9-CM) before 2015 and ICD-10-CM since 2016. Detailed records of drug prescriptions and procedure interventions and the comprehensiveness of the database, given that it covers an entire population, are the two main advantages of the NHIRD. However, the database does not contain laboratory data or examination reports. More information about the NHI program and NHIRD can be obtained from previous studies [22,23]. Before releasing data to researchers, any information in the database that can identify a particular patient or health care provider is encrypted to ensure privacy. This study was approved by the institutional review board of the Chang Gung Medical Foundation (Approval Number: 202100150B1).

### 2.2. Study Design

**Figure 1:**
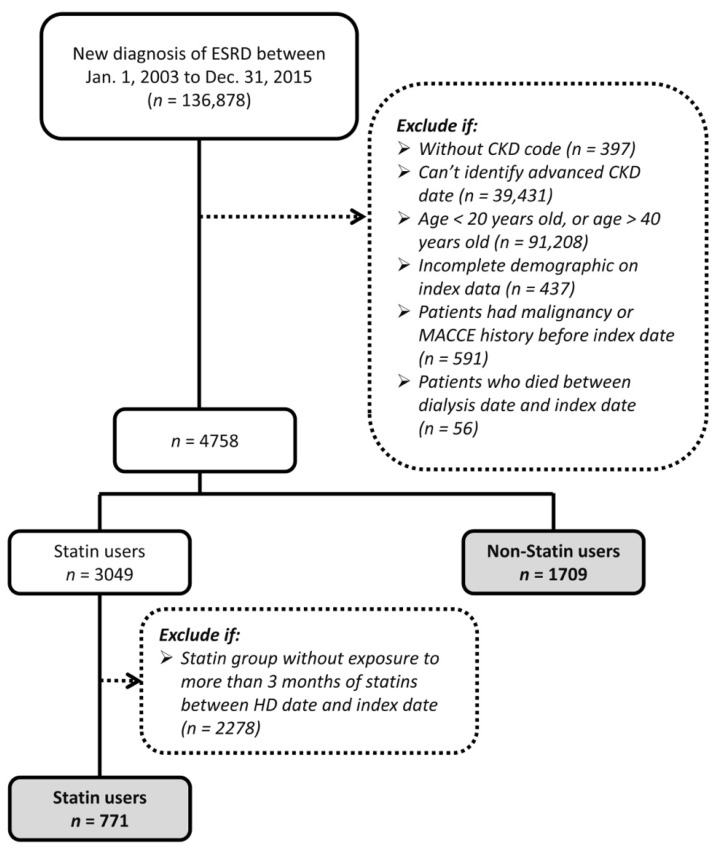
Flowchart showing the patient selection process.

We used data from the NHIRD to evaluate the association of mortality and CV events with the use of statins after dialysis initiation in young patients (age, 20–40 years) with ESRD. As shown in Figure 1, patients with newly diagnosed ESRD between 20 and 40 years of age and receiving dialysis treatment between 2003 and 2015 were identified. The 365th day after the start of dialysis was defined as the index date. Patients with a history of malignancy or MACCEs before the index date and patients who ended dialysis treatment (died or received kidney transplantation) between the start of hemodialysis and the index dates were excluded. Considering that the diet habit and long-term medications in patients with incident ESRD are gradually established over several months after commencing dialysis, we categorized all participants into two groups depending on whether they received any type of statin therapy for more than 90 days (statin group) or never received any statins (nonstatin group) in the first year after dialysis initiation. The follow-up period was from the index date (365th day after dialysis initiation) to the date of death, successful kidney transplantation, the independent occurrence of any of the study outcomes, or the end date of the study period (31 December 2017), whichever occurred first.

### 2.3. Covariates and Outcomes

The covariates in this study were age; sex; income level; residential urbanization level; comorbidities; Charlson comorbidity index (CCI) [24]; index date; frequency of hospitalization and outpatient visits, which may partially represent the patient’s health status; and medications. Comorbidities were determined if they were reported for more than two outpatient visits or one inpatient stay in the year before the index date. Medications were identified if a patient had received a prescription for more than 3 months between the dialysis and index dates.

We focused on the outcomes of all-cause mortality and MACCEs, which were defined as the composite of myocardial infarction, cardiogenic shock, new-onset heart failure, target vessel revascularization, malignant arrhythmia, stroke, and CV events. All-cause mortality was defined as the patient’s name appearing in the Taiwan Death Registry. Early death within the first year after dialysis initiation was mainly attributed to baseline comorbidities or nutritional status rather than receipt of statin treatment. Therefore, in this study, we only compared the results of interest in patients who underwent dialysis for more than 1 year, and the observation period began on the 365th day after dialysis initiation. MACCEs were diagnosed on the basis of the principal diagnosis upon hospitalization or during the emergency department visit. Diagnostic codes were ICD-9 codes before 2015 or ICD-10 codes from 2016 (Supplementary Materials Table S1), and most of them have codes been validated previously [25,26].

### 2.4. Statistical Analysis

Propensity score weighting was performed to simulate a randomized clinical trial by balancing baseline characteristics between study groups (statin and nonstatin groups). PSWs preserve the sample size of the original data, providing an appropriate estimate of the variation in the main impact and maintaining the specified type I error [27]. PSWs in the study groups were obtained using the generalized boosted model (GBM), which can automatically determine the best functions of covariates, including interactions or polynomial terms, to achieve the optimal balance in the study groups. Moreover, PSWs obtained using the GBM are not strongly affected by large weights [28].

All covariates listed in Table 1 were included in the GBM, except for the CCI, which was already a combination of other covariates. The absolute standardized mean difference (ASMD) rather than statistical tests was used to assess the balance of potential confounders between the groups at baseline (index date) because the balance is an attribute of the sample and not of the underlying population. ASMD ≤ 0.1 indicated that the difference in potential confounding factors between the groups was not significant, whereas ASMD < 0.2 indicated a small difference between the groups [28,29].

The incidence was calculated by dividing the total number of study results during the follow-up period by person-years at risk. The all-cause and CV mortality risks of the groups were compared using the Kaplan–Meier curve for univariate analysis and Cox proportional hazards model for multivariate analysis. The risks of MACCEs, acute myocardial infarction (AMI), and stroke were evaluated using competing risk analysis (subdistribution hazard function and cumulative incidence function) in which death during the follow-up period was regarded as a competing risk. We plotted the Kaplan–Meier curve for time to event outcomes. PSWs were re-estimated for each subgroup analysis to maintain a balance of covariates between the groups. *p* < 0.05 indicated statistical significance. All statistical analyses were performed using SAS 9.4 (SAS Institute Inc., Cary, NC, USA).

## 3. Results

### 3.1. Patient Characteristics

Data of 4758 young patients (age, 20–40 years) with new-onset ESRD receiving hemodialysis between 2003 and 2015 and who had ever received a diagnosis of dyslipidemia were extracted from the NHIRD (Figure 1). Of the patients, 771 had received statin therapy for more than 90 days between the dialysis initiation and index dates, and 1709 had never received any statin treatment. Those who received statin treatment less than 90 days were excluded. The baseline characteristics of the groups are presented in Table 1. The statin group had more female patients, patients with higher income, higher prevalence of hypertension and diabetes, higher comorbidity scores, greater use of certain medications (i.e., angiotensin-converting enzyme inhibitors or angiotensin receptor blockers, antihypertensive agents, aspirin (Plavix), insulin, and oral hypoglycemic agents), frequent outpatient visits, and higher probability of hospitalization than the nonstatin group before the PSW matching. After the PSW matching, most ASMD values were less than 0.1, and all ASMD values were less than 0.2, indicating that the clinical characteristics of the groups were well balanced.

### 3.2. Outcomes

**Figure 2:**
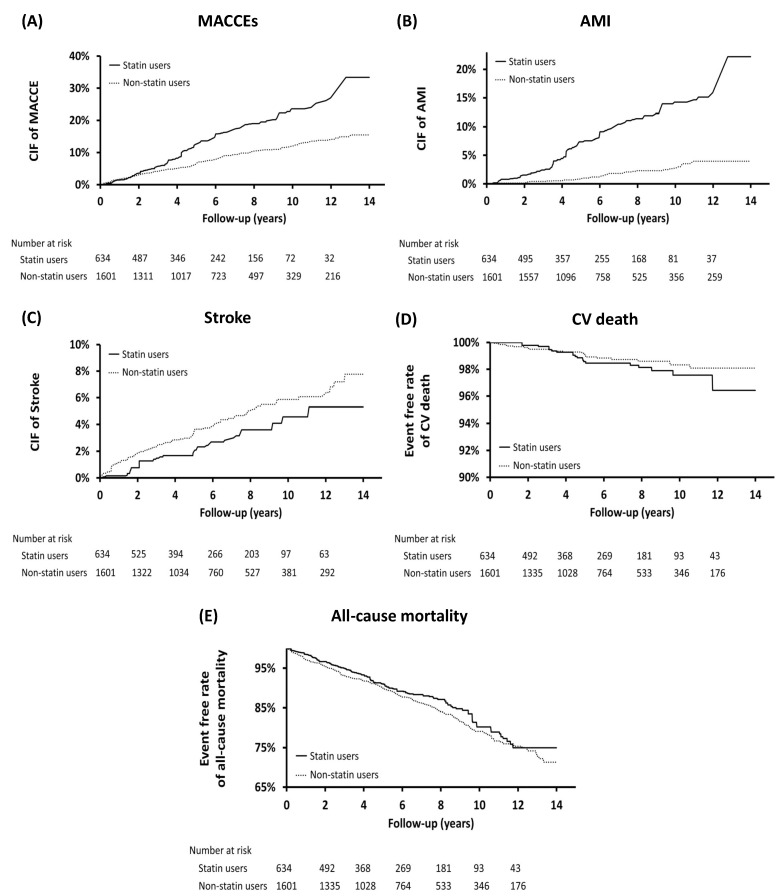
Cumulative incidence of (**A**) major cardiac and cerebrovascular events (MACCEs), (**B**) acute myocardial infarction [30], (**C**) stroke, (**D**) cardiovascular (CV) death, and (**E**) all-cause mortality. CIF: cumulative incidence function.

We aimed to assess whether statin treatment affects long-term outcomes in young patients with ESRD receiving permanent dialysis. The long-term outcomes are listed in Table 2. The statin group demonstrated a higher rate (per person-years) of MACCEs (2.65% vs. 1.44%, hazard rate (HR): 1.87, 95% confidence interval (CI): 1.43–2.45) and AMI (1.51% vs. 0.3%, HR: 5.34, 95% CI: 3.4–8.39) than the nonstatin group. The risk of all-cause mortality (1.96% vs. 2.28%, HR: 0.87, 95% CI: 0.66–1.14), CV mortality (0.23% vs. 0.17%, HR: 1.33, 95% CI: 0.57–3.08), and stroke (0.46% vs. 0.70%, HR: 0.66, 95% CI: 0.39–1.14) did not differ between the groups. The cumulative incidence of MACCEs, AMI, stroke, and CV and all-cause mortality is presented in Figure 2.

In order to avoid that the results of IPTW were largely affected by the data of patients with high weights, we have further adopted propensity score matching (PSM) to adjust (Supplementary Materials Table S2). After PSM, 619 people remained in the case and control groups, respectively. The result of the analysis is similar to the result of using the IPTW method, and the statin group still demonstrated a higher rate (per person-years) of MACCEs (2.73% vs. 1.53%, hazard rate (HR): 1.82, 95% confidence interval (CI): 1.29–2.58) and AMI (1.50% vs. 0.37%, HR: 4.16, 95% CI: 2.26–7.66) than the nonstatin group.

### 3.3. Subgroup Analysis

**Figure 3:**
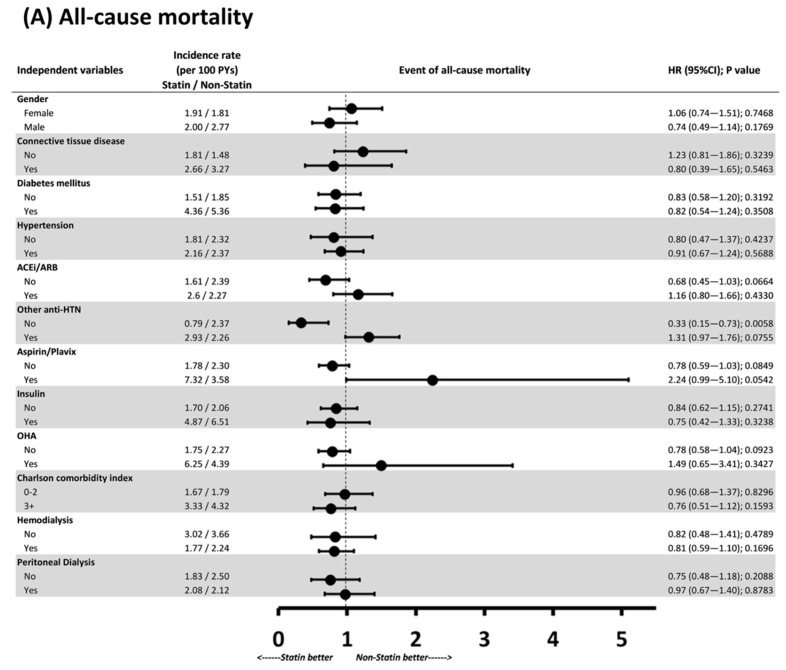

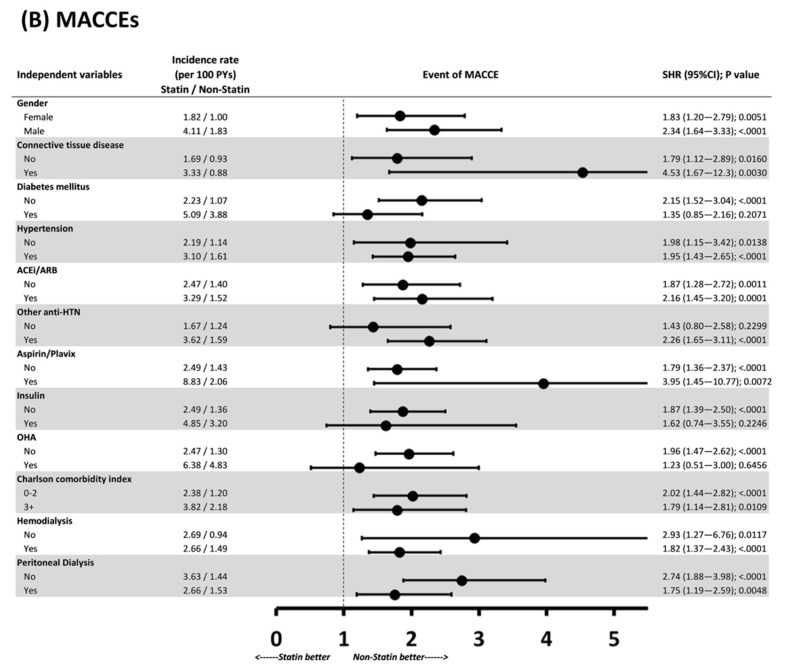
Subgroup analysis of (**A**) all-cause mortality and (**B**) MACCEs. PYs: person-years; HR: hazard ratio; SHR: subdistribution hazard ratio.

To determine whether the benefits of statin therapy were only seen in specific clinical conditions, we further conducted subgroup analysis of all-cause mortality and MACCE risk (Figure 3). Regarding all-cause mortality, statin therapy appeared to only have a protective effect in patients who were not taking any other antihypertensive drugs (HR: 0.33, 95% CI: 0.15–0.73, *p* = 0.0058); no significant difference was noted in other subgroups. Regarding MACCEs, the MACCE risk was higher in the statin group than in the nonstatin group except for the subanalyses of patients with diabetes and those not using other antihypertensive agents. The CIs displayed in the forest plots are wide, implying that the subgroup analyses were limited by the relatively small number of enrollees.

## 4. Discussion

The lack of benefits of statin therapy in patients receiving dialysis has been widely discussed. Experts argue that the lack of benefits can be attributed to long-term multiple comorbidities in the ESRD population and delayed initiation of statin treatment. However, the major finding of this 14-year retrospective cohort study, which analyzed data from a comprehensive nationwide database, is that lipid-lowering statin therapy fails to reduce the risks of MACCEs and all-cause and CV mortality even in young patients with ESRD, few comorbidities, and no history of MACCEs. The counterintuitive result of this study may complete the missing part of previous randomized controlled trials evaluating statin treatment after dialysis initiation and provide clinical evidence for better understanding the pathophysiology of CV events in ESRD populations.

By applying PSW to balance any possible confounders such as age, sex, hospitalization frequency, diabetes mellitus, hypertension, CCI, and commonly used medications, we demonstrated that compared with statin nonuse, statin use is associated with higher MACCE probability without reducing CV or all-cause mortality. The potential explanation for the disappointing finding is the different CV disease pathophysiologies in patients receiving dialysis compared with the general population. Importantly, the influences of mineral and bone metabolism disorder, volume overload, and control of hypertension may outweigh the importance of LDL level and atherosclerosis. First, studies have proven that higher average serum phosphorus concentration and daily calcium intake in patients receiving dialysis are related to coronary artery calcification and coronary artery disease [10,31]. In young adults, the imbalance of serum calcium and phosphate may be more severe than in older patients because of possible noncompliance with diet restrictions [32,33]. Atkinson et al. reported that fewer than one-quarter of young adults (age,18–24 years) could achieve the Kidney Disease Outcomes Quality Initiative (KDOQI)-recommended serum phosphorus and calcium levels [32]. Goodman et al. highlighted that the prevalence of coronary artery calcification, as measured using electron beam computed tomography, was as high as 88% among young patients (age, 20–30 years) receiving dialysis [31]. Second, in the young ESRD population, fluid restriction is another critical concern, and previous studies have reported their large fluid reduction during dialysis compared with older populations [33,34]. Repeated myocardial stunning induced by large fluid reduction during dialysis may eventually result in sudden CV death. Lastly, along with fluid overload and poor compliance with treatment regime, hypertension is the leading comorbidity in the younger population [20,35]. Importantly, compared with older patients with ESRD, who typically have multiple comorbidities, our study participants had considerably higher prevalence of hypertension than prevalence of other chronic diseases. Statin treatment cannot correct complications associated with long-term, poorly controlled hypertension, such as LVH and arrhythmias. In a database study, it is difficult to comprehensively analyze all factors due to a lack of detailed information, such as diet habits, fluid reduction during dialysis, and calcium and phosphate balance. However, we found that statin treatment could not ameliorate the nonatherosclerotic CV risks in the ESRD population, resulting in a similar mortality rate between the statin and nonstatin groups.

The higher MACCE and AMI risks in this study’s statin group could not be explained and can be considered a chance finding. In an observational study of patients receiving renal replacement therapy, statin use was associated with a higher baseline coronary artery calcification (CAC) score (regardless of age, sex, and diabetes) and faster progression of the CAC score compared with no statin use in a longitudinal evaluation [30]. Moreover, several randomized studies involving patients without kidney disease have demonstrated that statins promote coronary atheroma calcification independent of their plaque-regressive effects, and which also did not correlate with a greater risk of CV events [36,37]. Statins change the composition of the coronary atheroma, thicken the fibrous cap, replace the central lipid pool with calcification and fibrosis, decrease plaque volume, and reduce inflammation [38,39]. These shifts in plaque components with increased calcium content stabilize vulnerable plaques, reducing the risk of rupture. However, the plaque-stabilizing effect of increased coronary artery calcification in the general population may not be considered benign in the ESRD dialysis population. In patients receiving dialysis, obvious calcium deposits in the vascular intimal and media layers and aortic and mitral valves are highly prevalent due to pathophysiological changes. The uremic vasculopathy and cardiomyopathy contribute to the excessively high mortality rate from cardiac causes. Overall, statin-induced calcification may aggravate the already severely elevated CV risk in the uremic milieu. Certain hypotheses regarding the procalcifying effect of statins have also been proposed recently. Statins not only lower lipid production by inhibiting HMG CoA reductase, the rate-controlling enzyme of the mevalonate pathway responsible for the synthesis of cholesterol and other isoprenoids but also impair vitamin K2 generation in vitro by affecting the metabolism of isoprenoids [30,40]. Vitamin K is essential for the activity of vascular calcification inhibitors such as matrix Gla28 protein. Compared with healthy individuals, patients receiving hemodialysis have poor overall vitamin K status due to low intake [41], and vitamin K supplementation has been proposed to arrest the progression of vascular calcification [42,43]. Thus, statins may accelerate vascular calcification in patients receiving dialysis by further depleting the vascular vitamin K2 level. Further studies are warranted to prove whether the procalcifying effect of statins abrogates the potential favorable effects of LDL cholesterol reduction in patients receiving dialysis.

This study has certain limitations. First, data for analysis were retrieved from the NHIRD, which does not contain laboratory information, including lipid profiles and levels of hemoglobin, creatinine, phosphate, calcium, and albumin. Although previous large-scale RCTs have proved the significant LDL-lowering effect of statin treatment. The lack of LDL-cholesterol level information in NHIRD, which thus makes us unable to verify whether the statin group could truly achieve a lower LDL level than the nonstatin group, is the main limitation of this study. Further large-scale researches with comprehensive laboratory data are warranted to validate our findings. Second, although the PSW analysis included the most relevant confounders, all residual factors could not be eliminated because of the observational nature of the study, and this may have biased the results. Third, because of the number of enrollees and study design, evaluating the beneficial effect across different statin therapies and statin dosages was beyond the scope of this study.

## 5. Conclusions

In conclusion, statin therapy did not satisfactorily improve all-cause and CV mortality in young adults with ESRD receiving dialysis, even in those without a history of MACCEs. Therefore, we suggest prevention strategies—including lifestyle modification, exercise, dialysis therapy adherence, diet and fluid restriction, and early transplantation—instead of primarily focusing on lipid control to reduce the CV risk in young adults with ESRD.

## Figures and Tables

**Table 1:** Baseline characteristics of the study population before and after PSW matching.

	Before PSW			After PSW		
	Statin Users (*n* = 771)	Nonstatin Users (*n* = 1709)	ASMD	Statin Users (*n* = 635)	Nonstatin Users (*n* = 1601)	ASMD
Age (years)	33.81 ± 5.03	32.89 ± 5.67	0.1713	33.42 ± 4.72	33.1 ± 5.33	0.0645
Male, *n* (%)	300 (38.91)	990 (57.93)	0.3876	289 (45.56)	850 (53.08)	0.1509
Urb, *n* (%)			0.1439			0.0493
Urban	221 (28.66)	431 (25.22)		180 (28.36)	416 (25.96)	
Suburban	257 (33.33)	519 (30.37)		196 (30.98)	495 (30.94)	
Rural	293 (38.00)	759 (44.41)		258 (27.19)	690 (43.10)	
Occupation, *n* (%)			0.1245			0.0958
Dependent	80 (10.38)	230 (13.46)		72 (11.32)	202 (12.62)	
Civil servants	20 (2.59)	47 (2.75)		15 (2.37)	43 (2.68)	
Nonmanual worker	230 (29.83)	444 (25.98)		181 (28.37)	436 (27.26)	
Manual worker	256 (33.20)	549 (32.12)		202 (31.85)	514 (32.10)	
Other	185 (23.99)	439 (25.69)		163 (25.83)	406 (25.34)	
Income (NTD), *n* (%)			0.2232			0.0777
Dependent	80 (10.38)	230 (13.46)		72 (11.32)	202 (12.62)	
<17,280	219 (28.40)	556 (32.53)		195 (30.70)	513 (32.08)	
17,280–21,008	156 (20.23)	374 (21.88)		136 (21.52)	338 (21.13)	
21,009–33,229	187 (24.25)	336 (19.66)		136 (21.52)	328 (20.48)	
>33,300	129 (16.73)	213 (12.46)		95 (14.94)	219 (13.69)	
Comorbidities, *n* (%)						
Atrial fibrillation	0 (0.00)	3 (0.18)	0.0593	0 (0.00)	4 (0.23)	0.0674
Chronic pulmonary disease	20 (2.59)	35 (2.05)	0.0363	15 (2.30)	32 (1.97)	0.0228
Connective tissue disease	94 (12.19)	140 (8.19)	0.1325	66 (10.42)	148 (9.27)	0.0386
Diabetes mellitus	299 (38.78)	185 (10.83)	0.6841	135 (21.34)	259 (16.15)	0.1332
Heart failure	39 (5.06)	96 (5.62)	0.0249	28 (4.43)	84 (5.23)	0.0370
Hypertension	582 (75.49)	1019 (59.63)	0.3438	435 (68.62)	1013 (63.29)	0.1125
Liver cirrhosis	3 (0.39)	29 (1.70)	0.1290	<3 (<0.45)	22 (1.36)	0.1290
Peripheral arterial disease	7 (0.91)	26 (1.52)	0.0560	4 (0.65)	21 (1.31)	0.0674
Polycystic kidney disease	5 (0.65)	22 (1.29)	0.0653	9 (1.39)	18 (1.13)	0.0234
Charlson comorbidity index,	2.25 ± 1.1	1.88 ± 0.98	0.3592	1.99 ± 0.91	1.94 ± 0.96	0.0580
Medications, *n* (%)						
ACEi/ARB	438 (56.81)	592 (34.64)	0.4565	287 (45.21)	634 (39.58)	0.1141
Other anti-HTN	598 (77.56)	845 (49.44)	0.6107	398 (62.76)	900 (56.24)	0.1331
Aspirin/Plavix	64 (8.30)	58 (3.39)	0.2103	30 (4.81)	58 (3.63)	0.0586
Insulin	151 (19.58)	73 (4.27)	0.4862	64 (10.12)	111 (6.96)	0.1131
OHA	123 (15.95)	49 (2.87)	0.4599	50 (7.81)	73 (4.59)	0.1339
No. of outpatient visits in the previous year	33.68 ± 14.70	29.51 ± 12.40	0.3061	31.00 ± 11.69	30.32 ± 12.38	0.0562
Hospitalizations in the previous year, *n* (%)	339 (43.90)	639 (37.97)	0.1342	257 (40.60)	620 (38.72)	0.0384

PSW, propensity score weighting; ASMD, absolute standardized mean difference; ACEi/ARB, angiotension converting enzyme inhibitor/angiotension receptor blocker; HTN, hypertension; OHA, oral hypoglycemic agent.

**Table 2:** Follow-up outcomes of young adult patients with ESRD according to their use of statins.

	Statin Users			Nonstatin Users			Statin Users vs. Nonstatin Users
	No. of Event	Person-Years	Incidence Rate	No. of Event	Person-Years	Incidence Rate	SHR/HR (95%CI); *p* Value
MACCE ^a^	87	3263.61	2.65 (2.09–3.21)	138	9608.12	1.44 (1.20–1.68)	1.87 (1.43–2.45); <0.0001
AMI	51	3376.31	1.51 (1.09–1.92)	30	9940.81	0.30 (0.19–0.41)	5.34 (3.40–8.39); <0.0001
Stroke	16	3475.96	0.46 (0.24–0.69)	68	9815.81	0.70 (0.53–0.86)	0.66 (0.39–1.14); 0.1368
CV death	8	3524.99	0.23 (0.10–0.44)	17	10,025.6	0.17 (0.09–0.25)	1.33 (0.57–3.08); 0.5132
All-cause mortality	69	3524.99	1.96 (1.50–2.42)	229	10,025.6	2.28 (1.99–2.58)	0.87 (0.66–1.14); 0.3058

^a^: Any of myocardial infarction, cardiogenic shock, new-onset heart failure, malignant arrhythmia, and cerebrovascular events; AMI: acute myocardial infarction; CI: confidence interval; CV: cardiovascular; HR: hazard ratio; SHR: subdistribution hazard ratio.

## Data Availability

Not applicable.

## Supplementary Materials

The following are available online at https://www.mdpi.com/article/10.3390/jcm10102097/s1, Table S1: ICD9-code and ICD10-code used in this study; Table S2: Follow-up outcomes (After propensity score matching).
