# Supplementary material for: Does Statin Therapy Reduce the Risks of Mortality and Major Adverse Cardiac and Cerebrovascular Events in Young Adults with End-Stage Renal Disease? Population-Based Cohort Study
Source: J Clin Med. 2021 May 13;10(10):2097. doi: 10.3390/jcm10102097 (PMC8152985; doi:10.3390/jcm10102097)
Supplement: Supplementary file 1 [file jcm-10-02097-s001.zip › jcm-1198115-supplementary.docx]

**Supplementary Materials:**

**Table S1.** ICD9-code and ICD10-code used in this study.

| **Disease** | **ICD9-code** | **ICD10-code** |
| --- | --- | --- |
| Chronic kidney disease (CKD) | 016.0, 042, 095.4, 189, 223, 236.9, 250.4, 271.4, 274.1, 403-404, 440.1, 442.1, 446.21, 447.3, 572.4, 580-589, 590-591, 593, 642.1, 646.2, 753, 984 | B20, A52.75, C64-68, D30, D41, E11.29, E74.8, M10.30, N20, I70.1, I72.2, M31, I77.3, K76.7, N00-08, N10-15, N18, N28.83, N28.81, N28.1, N28.9, O10.41-43, O12.14, O26.831-839, Q60-4 |
| End-stage renal disease (ESRD) | 585 | N186 |
| Kidney transplant | 55.69 | Z94.0 |
| **Comorbidities** |  |  |
| Hypertension | 401-405 | I10-I16 |
| Dyslipidemia | 272 | E78 |
| Liver cirrhosis | 571.2, 571.5, 571.6 | K70.30-K70.32, K74.5, K74.60, K71.7 |
| Connective tissue disease | 710, 714, 728 | M30-M36 |
| Atrial fibrillation | 427.31 | I48(exclude I48.3 and I48.9) |
| Peripheral arterial disease (PAD) | 440.2, 440.3, 440.8, 440.9, 443, 444.22, 444.8, 444.9 | I70.2-I70.9, I73.9 |
| Chronic pulmonary disease | 491-496 | J41-J47 |
| Diabetes Mellitus | 249-250 | E08-E13 |
| Heart failure | 428 | I50 |
| Polycystic kidney disease | 753.12 | Q61.2 |
| **Major adverse cardiac/cerebrovascular events (MACCEs)** |  |  |
| Cardiogenic shock | 785.51 | R57.0 |
| Heart failure (HF) | 428 | I50 |
| Malignant dysrhythmia | 426.0, 426.12-426.13, 426.51-426.52, 426.54, 427.1, 427.4, 427.41-427.42, 427.5 | I44.0-3, I45.2, I45.3, I46.9, I47.2, I49.0-3 |
| Myocardial infarction | 410 | I21, I22 |
| Stroke | 430-437 | I60-64, G45.0, G45.1, G45.4, G45.8, I67 |

**Table S2:** Follow-up outcomes (After propensity score matching).

| After PSM (1:1) | | | | | | | |
| --- | --- | --- | --- | --- | --- | --- | --- |
|  | **Statin users** | | | **Non-Statin users** | | | **Statin users vs.**  **Non-Statin users** |
|  | **No. of event** | **Person-years** | **Incidence rate** | **No. of event** | **Person-years** | **Incidence rate** | **SHR/HR(95%CI); P value** |
| MACCE ^a^ | **85** | **3107.87** | **2.73(2.15-3.32)** | **52** | **3391.32** | **1.53(1.12-1.95)** | **1.82(1.29-2.58); 0.0007** |
| AMI | **48** | **3204.54** | **1.50(1.07-1.92)** | **13** | **3503.07** | **0.37(0.17-0.57)** | **4.16(2.26-7.66); <.0001** |
| Stroke | **16** | **3296.34** | **0.49(0.25-0.72)** | **24** | **3468.87** | **0.69(0.42-0.97)** | **0.71(0.37-1.33); 0.2806** |
| CV death | **9** | **3327.64** | **0.27(0.12-0.51)** | **5** | **3531.96** | **0.14(0.05-0.33)** | **1.95(0.65-5.82); 0.2318** |
| All-cause mortality | **77** | **3327.64** | **2.31(1.80-2.83)** | **94** | **3531.96** | **2.66(2.12-3.20)** | **0.88 (0.65-1.19); 0.3941** |
| ^a^: Any of myocardial infarction, cardiogenic shock, new-onset heart failure, malignant arrhythmia, and cerebrovascular events; AMI: acute myocardial infarction; CI: confidence interval; CV: cardiovascular; HR: hazard ratio; SHR: subdistribution hazard ratio. | | | | | | | |
|  |  |  |  |  |  |  |  |
